# A review of the best method of leg wound closure following open harvesting of the long saphenous vein for coronary artery bypass grafting

**DOI:** 10.1016/j.amsu.2021.102855

**Published:** 2021-09-13

**Authors:** Pedram Panahi, Ali Adeeb Ilyas, Clinton Lloyd, Adrian Marchbank, Jonathan Unsworth-White

**Affiliations:** Department of Cardiothoracic Surgery, University Hospitals Plymouth NHS Trust, Derriford Road, Plymouth, PL6 8DH, United Kingdom

**Keywords:** Evidence-based medicine, Coronary artery bypass graft, Coronary revascularization, Long saphenous vein, Great saphenous vein, Closure method, Single layer closure, Double layer closure, Multiple layer closure

## Abstract

Uncertainty exists around the optimal method of leg wound closure following open long saphenous vein harvesting in adults undergoing coronary artery bypass graft surgery (CABG). Such is evident from the variety observed in the closure approach utilised. Consequently, a best evidence topic in cardiac surgery was written according to a structured protocol. The question addressed was ‘following open long saphenous vein harvesting in adults undergoing CABG, is single-layer leg wound closure superior to multiple-layer closure in terms of post-operative complications encountered? ‘. Altogether 382 papers on Ovid Embase and Ovid Medline, 301 papers on PubMed and 11 papers on the Cochrane database were found using the reported search. From the screened articles, 6 represented the best evidence to answer the clinical question. The authors, journal, date and country of publication, patient group studied, study type, relevant outcomes and results of these papers are tabulated. We conclude that the best method of leg closure following open saphenous vein harvesting for CABG is single-layer cutaneous closure. The use of a suction drain to eliminate the dead space should be considered on a case-to-case basis by the lead operating surgeon with the patient's characteristics and their own expertise in mind.

## Highlights

• Uncertainty exists around the best closure method after open harvesting of the long saphenous vein for CABG in adults.
• Various options include single-layer and multiple-layer closure.
• Therefore, we conducted a literature review to identify the best closure technique.
• We conclude that the best method is single-layer cutaneous closure.
• The use of a suction drain to eliminate the dead space should be considered.

## 1 Introduction

Leg wound complications following coronary artery bypass graft surgery (CABG) are a major cause for morbidity requiring further invasive interventions [1]; a large-scale study by Paletta et al. reported an average lower extremity complication rate of 4.1% which conforms with the range observed in other literature findings [[1], [2], [3], [4]]. As such, it is important to study every surgical aspect to identify methods of minimising the complication rate where possible in addition to making economic and time savings. Endoscopic vein harvesting of the long saphenous vein (LSV) is gaining popularity over the open approach owing to its lower complication rate [5]; however, several centres continue to use the open technique due to factors such as the harvest time, learning curve and cost. In this study, we have focused on the open technique. Traditionally, when employing the open approach to harvesting the LSV as a conduit for CABG, a double-layer closure technique is used where the subcutaneous tissue is closed first followed by cutaneous closure. Here, we review the best evidence available to determine whether the multiple-layer approach should be replaced by a single-layer cutaneous closure. This best evidence topic was constructed according to a structured protocol; this is fully described by the International Journal of Surgery [6].

## 2 Clinical scenario

A 74-year-old patient with a background of type 1 diabetes mellitus initially presented to the chest pain clinic with angina. Further investigations were carried out including percutaneous angiography which revealed severe triple vessel disease of the coronary arteries. Echocardiography found no valvular pathology and a left ventricular ejection fraction of 30–35%. The case was discussed at the multidisciplinary meeting and CABG was recommended. You discuss the choice of conduit with the patient. The patient who is of a surgical background enquires further about the outcomes encountered when using a single-layer leg wound closure compared with multiple-layer closure. Unsure of the best closure technique, you resolve to check the literature for evidence.

## 3 Three-part question

In [adults undergoing coronary artery bypass graft surgery], which method of leg wound closure following open long saphenous vein harvesting [single-layer versus multiple-layer closure] is superior in terms of [length of admission and post-operative complications] encountered?

## 4 Search strategy

The search strategy outlined below was utilised and where possible the results were limited to English articles, Persian articles and human studies. In addition, the reference lists of the screened articles were reviewed.

Medline 1946 to May 2021 and Embase 1974 to May 2021 using the OVID interface:

[(single layer) OR (single-layer) OR (double layer) OR (double-layer) OR (multiple layer) OR (multiple-layer) OR (multi layer) OR (multi-layer) OR (unilayer) OR (uni-layer) OR (bilayer) OR (bi-layer) OR (closure)] AND [(bypass) OR (cardiac surgery) OR (cardiothoracic) OR (cardiac)] AND [Saphenous]

Medline using the PubMed interface:

[(single layer) OR (single-layer) OR (double layer) OR (double-layer) OR (multiple layer) OR (multiple-layer) OR (multi layer) OR (multi-layer) OR (unilayer) OR (uni-layer) OR (bilayer) OR (bi-layer) OR (closure)] AND [(bypass) OR (cardiac surgery) OR (cardiothoracic) OR (cardiac)] AND [Saphenous]

Cochrane Database:

Saphenous vein, layer

## 5 Search outcome

**Fig. 1:**
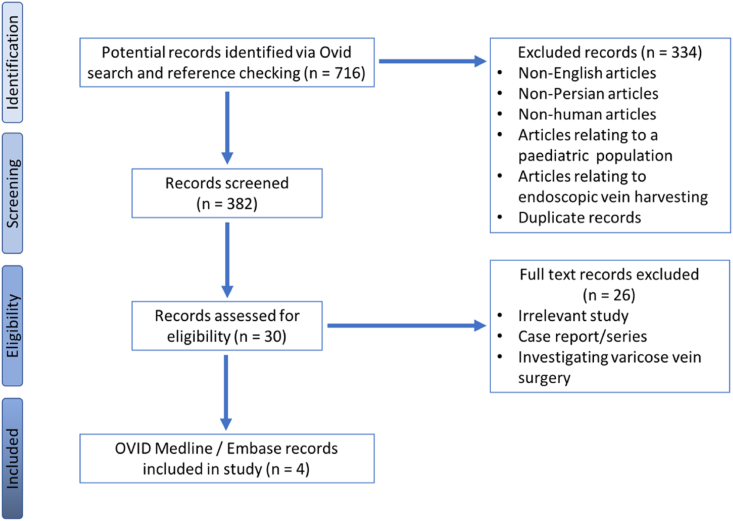
PRISMA flow chart for Ovid search (PubMed and Cochrane flow chart not included in this figure).

382 papers on Ovid Embase and Ovid Medline, 301 papers on PubMed and 11 papers on the Cochrane database were found using the reported search and screened. From these, 6 papers were identified that provided the best evidence to answer the question determining the optimal leg wound closure technique following open LSV harvesting in adults undergoing CABG. These are presented in Appendix 1. An example of the screening and eligibility assessment process for the search results obtained from the Ovid interface is detailed in Fig. 1.

## 6 Results

The results of this article are tabulated in Appendix 1 which contains a review of the most relevant and highest quality evidence available assessing the best method of leg wound closure after harvesting the LSV for CABG. This table is structured according to the guidance by the International Journal of Surgery [6] highlighting key results, statistical analysis and study limitations.

## 7 Discussion

In 2011, a randomised controlled trial by Siddiqi et al. compared single-layer closure of the leg over a suction drain (with drain removal after 48 hours) against double-layer closure following extraction of the LSV for CABG [7]. The harvesting of the vein was performed by a single surgeon and the patients were followed up until two weeks after discharge. The ASEPSIS score was used to assess the wound in this study; this scoring method was first described in a study published in the Lancet by Wilson et al. on cardiac surgery patients [8] and has been shown to be a reliable method of wound assessment [9]. The ASEPSIS score allocates points for the following: need for Additional treatment, Serous discharge, Erythema, Purulent exudate, Separation of deep tissues, Isolation of bacteria, and the duration of inpatient Stay. In the study by Siddiqi et al. the mean ASEPSIS score of both single- and double-layer groups were within the satisfactory healing category; however, a statistically significant lower ASEPSIS score was observed in the single-layer group compared with the double-layer group. Furthermore, a smaller percentage of complications were encountered in the single-layer group. Consequently, it was concluded that single-layer closure of the leg wound should be the method of choice.

Tiryakioglu et al. conducted a randomised controlled trial in 2010 comparing single-layer closure with double-layer closure following saphenectomy for CABG [10]. Several aspects of the leg wound were assessed up to 2 months post-operatively. There were no statistically significant differences between the two groups in terms of the demographics, operative time, number of grafts and hospitalisation period. Whilst it was found that up to the point of 1 week post-discharge, the incidence of haematoma was higher in the single-layer group, this was not statistically significant. On the other hand, the single-layer group demonstrated a statistically significant lower incidence of infection, oedema, numbness and number of legs with associated complaints. As such, it was reasonably concluded that single-layer closure should be the favoured method.

In 2006, a randomised controlled trial by Stenvik et al. investigated single-layer leg wound closure against double-layer closure and further investigated the impact of the operating practitioner harvesting the vein (rotational surgical residents against one dedicated experienced physical assistant) [11]. Whilst a lower incidence of infection was observed in the single-layer group, this was not statistically significant. Of note is the fact that there was a significant lower infection rate in the group operated on by one dedicated physical assistant when compared to the group operated on by surgical residents. In terms of closure technique, similar findings were reported in a study by Teebken et al. who observed no significant difference between the two closure methods when considering haematoma formation, length of hospital stay, infection and wound dehiscence; this article by Teebken et al. has not been tabulated in the present study because it is not available in English [12].

Zafar et al. conducted a randomised controlled trial in 2005 to compare single-layer leg closure over a suction drain (with drain removal after 24 hours) against double-layer closure following saphenectomy for CABG [13]. The legs were reviewed every 48 hours until discharge and at 6 weeks in the outpatient clinic. The randomisation method of minimisation and the statistical analysis are clearly defined. The ASEPSIS score, used to assess the wound in this study, suggested satisfactory wound healing in both the single-layer and double-layer group. However, keeping in mind that a lower ASEPSIS score indicates a better outcome, the single-layer group had a statistically significant lower ASEPSIS score; this finding was maintained when the diabetic and non-diabetic subgroups were analysed separately. Considering the statistically significant lower ASEPSIS score and incidence of donor leg oedema as well as the quoted decreased dead space due to haematoma evacuation and reduced tissue handling, the single-layer technique of leg wound closure with a drain was determined to be the superior method.

A randomised controlled trial by Nouraei et al. in 2010 concluded that single-layer closure of the LSV donor site with a haemovac drain is superior to multiple-layer closure in several aspects at day 2, 14 and 21 post-CABG [14]. The study was limited to those who were non-urgent, BMI less than 30, under 70 years of age and non-diabetic patients. The authors specify that the multiple-layer closure was achieved by closing the “skin, subcutaneous tissue, subcutaneous fat and scarpa's fascia”. On day 2 post-operatively, the only statistically significant difference was observed in the lower occurrence of haematomas in the single-layer group. Throughout the following two timepoints, there was a statistically significant lower occurrence of all complications in the single-layer group, apart from saphenous nerve paraesthesia on day 14 and day 21 as well as skin necrosis on day 21 whereby the lower occurrence of these complications in the single-layer group was not statistically significant.

In contrast, a study by Nair et al. assessing the cutaneous sensation in a randomised controlled trial (n = 50) comparing single-layer interrupted sutures without a drain to multiple-layer closure, excluding those with diabetes and peripheral arteriopathy, found less neurological complications and better sensory perception recovery in the single-layer closure group [15]. Apart from gender, no other demographics were disclosed. Wound infection or seroma was not observed in either group. Three patients were excluded due to injury to the branches of the long saphenous nerve. The lower incidence of anaesthesia, paraesthesia and pain in the single-layer group was statistically significant at 1 and 6–8 weeks post-operatively, but not at day two. There was also a lower incidence of neurologic complications in the single-layer group at 14–18 months, though this was not statistically significant. The observed neurological differences were attributed to nerve compression secondary to the subcutaneous sutures.

A randomised controlled trial by El Gamel et al. in 1994 compared single-layer closure against double-layer closure following saphenectomy for CABG [16]. In the first subset, the patients were recruited from the UK and the LSVs were harvested from below the knee of both legs of the same patient; therefore, the patient acted as their own control whereby one leg was randomly assigned to a fat stitch (double-layer closure) and the contralateral leg of the same patient to no fat stitch (single-layer closure). In the second subset, the patients were recruited from the USA and the LSV was harvested from the thigh of one leg (above the knee). Wounds were observed until discharge and later reviewed in the outpatient clinic. The interesting approach for the first subset was unique to this study allowing for controlling of several confounding factors except unequal peripheral arterial disease and such patients were appropriately excluded. However, an explanation is not provided as to why this approach was not used for the second subset. El Gamel et al. concluded that double-layer closure takes longer and may increase skin edge necrosis requiring plastic surgical intervention. Considering the surgical time and comparable complication (Haematoma/infection) rate between single- and double-layer closure, it was suggested that the use of a fat suture is not necessary and as such its use should be discontinued.

The discussions of this study are limited by the weaknesses of the included articles which are highlighted in the comments section of the table in Appendix 1. For instance, it is important to note that the inconsistency in wound healing descriptors makes comparisons between studies more challenging. The ASEPSIS scoring system has been validated as a reproducible method to quantify wound healing and is recommended for future studies in this field.

## 8 Clinical bottom line

Taking into account the above discussed articles representing the best evidence topics available, it is evident that the best method of leg wound closure following LSV harvesting for CABG is single-layer cutaneous closure. Some of the discussed studies combined single-layer closure with the use of a suction drain; as such, the use of a suction drain to eliminate the dead space should be considered on a case-to-case basis by the lead operating surgeon with the patient's characteristics and their own expertise in mind.

## Ethical approval

Not required.

## Funding

None.

## Author contribution

• Mr. Pedram Panahi, MBBS, MRes (Distinction), PGCert (Clinical Education), MRCS: Generated research proposal, conducted literature search, data collection, data entry into table and manuscript write up.
• Dr. Ali Adeeb Ilyas, MBBCh, BAO: Reviewed literature search methodology, data collection and data entry into table.
• Mr. Clinton Lloyd, MBChB, FRCS(CTh): Reviewed research proposal and manuscript.
• Mr. Adrian Marchbank, BSc, MBBS, FRCS (CTh, McCormack Medal): Reviewed research proposal and manuscript.
• Mr. Jonathan Unsworth-White, BSc (First Class Hons), MBBS (Double Hons), FRCS (CTh, McCormack Medal): Supervising consultant, generated research proposal and reviewed manuscript.

## Consent

Not required.

## Registration of research studies

Not required.

1. Name of the registry:
2. Unique Identifying number or registration ID:
3. Hyperlink to your specific registration (must be publicly accessible and will be checked):

## Guarantor

Pedram Panahi

## Declaration of competing interest

None to declare.

## Figures and Tables

**Table:** 

Article	Author, date, journal, country and study type (Level of Evidence)	Patient group	Outcomes	Key results	Comments
Prospective comparative study of single-layer versus. Double-layer closure of leg wounds after long saphenous vein harvest in coronary artery bypass graft operations [7]	Siddiqi et al. (2011)	n = 77 - Single layer (SL) = 52 - Double layer (DL) = 25	Length of incision	- SL = Median (52 cm) and mean (52.6 cm) - DL = Median (54 cm) and mean (58.5 cm)	Randomisation method not delineated, but method of statistical analysis clearly defined. No conflict of interest reported. Non-significant discrepancy was observed in the gender of patients (58 male and 19 female patients), method of closure (52 by SL and 25 by DL) and proportion of diabetics (61.53% of SL subgroup and 41.6% of DL subgroup). Statistically significant discrepancy was observed in the proportion of those with renal failure (7.6% of SL subgroup and 33.3% of DL subgroup - *p* = 0.012). The above discussed discrepancies may be a source of selection bias in this study. NB. a lower ASEPSIS score indicates better healing.
			Infection	- Mean ASEPSIS score in all patients - 4.038 in SL and 9.467 in DL - *p* < 0.001 - Mean ASEPSIS score in diabetics - 3.69 in SL and 13.2 in DL - Reported as statistically significant, though level of significance not mentioned. - Infective wound characteristic - observed in 0% of the SL and 3.33% of the DL group - Statistical significance not measured.	
	Journal of Thoracic Disease, Oman, Prospective randomised trial (Level 1b)		Oedema/erythema/haematoma/seroma	- Proportion of SL group affected - Serous discharge (28.8%), inflammation (23.07%) and oedema (23.07%). - Proportion of DL group affected - Serous discharge (46.6%), inflammation (46.6%) and oedema (53.3%). No statistical significance assessment of the difference in these within the article.	
			Length of admission	- SL = 10.67 days - DL = 11 days	
			Other	- Proportion of SL group affected by pain (44.2%). - Proportion of DL group affected by pain (73.33%). The difference is specified as statistically significant, but the exact *p* value not mentioned.	
Unilayer closure of saphenous vein incision lines is better than bilayer closure [10]	Osman Tiryakioglu et al. (2010)	n = 79 (Total recruited number was reduced from 82 following exclusion of 3 patients due to inpatient mortality). - Single layer (SL) = 41 - Double layer (DL) = 38	Length of incision	- SL = 40 ± 5.5 cm (Median) - DL = 43 ± 6 cm (Median) Difference not statistically significant.	Randomisation method not defined, but method of statistical analysis clearly delineated. No declaration of conflict of interest. No statistically significant difference in the demographics of the two groups. Further, no differences in operative time, number of grafts or hospitalisation period. To assess oedema, a measuring tape mounted on a fixed surface (the Leg-O-meter) was used which has been found to be >97% reliable in a study by Berard et al. [17]. However, there is risk of bias due to the fact that there is no mention of who undertook the measurement using a Leg-O-meter. Haematoma/infection was assessed by clinical examination. It is reported that the patients' medical history was used to evaluate pain and numbness which is subjective, and therefore introduces risk of confirmation bias; additionally, it is not explained whether the medical history was obtained directly from the patient or indirectly from their medical notes.
			Infection	- Findings up to 1 week post discharge - SL (4.8%) and DL (21%) - *p* = 0.033 - Findings at 2nd post-operative month - SL (0%) and DL (5.2%) - *p* = 0.05	
	The Open Cardiovascular Medicine Journal, Turkey, Prospective randomised trial (Level 1b)		Oedema/erythema/haematoma/seroma	Oedema - Findings up to 1 week post discharge - SL (7%) and DL (26%) - *p* = 0.023 - Findings at 2nd post-operative month - SL (2.4%) and DL (15.7%) - *p* = 0.04 Haematoma - Findings up to 1 week post discharge - SL (14%) and DL (5.2%) - NS - Findings at 2nd post-operative month - SL (0%) and DL (0%) - NS Haematoma incidence findings were not statistically significant (NS).	
			Length of admission	- SL = 7.3 ± 3 days - DL = 8 ± 2 days Difference is not statistically significant.	
			Other	Pain - Findings up to 1 week post discharge - SL (14%) and DL (21%) - NS - Findings at 2nd post-operative month - SL (2.4%) and DL (10.4%) - NS Numbness - Findings up to 1 week post discharge - SL (4.8%) and DL (31.5%) - *p* = 0.002 - Findings at 2nd post-operative month - SL (0%) and DL (26%) - *p* = 0.001 Number of patients with leg complaints - Findings up to 1 week post discharge - SL (14%) and DL (21%) - NS - Findings at 2nd post-operative month - SL (2.4%) and DL (15.7%) - *p* = 0.04	
Effect of subcutaneous suture line and surgical technique on wound infection after saphenectomy in coronary artery bypass grafting: A prospective randomised study [11]	Stenvik et al. (2006)	n = 239 (Total recruited number was reduced from 243 following exclusion of 4 patients from group A). This sample was constituted from two subgroups: Group A (Test group, total = 119) were all operated on by one physical assistant and the patients randomised to either single layer (SL = 59 patients) or double layer (DL = 60 patients). Group B (Control group, total = 120) were all operated on by rotational surgical residents.	Length of incision	Not assessed.	Randomisation method not defined. However, the statistical analysis method is clearly delineated. No declaration of conflict of interest. The reasoning behind removal of 4 patients from group A is clearly accounted for (1 death and 3 conversions to off pump surgery). Some of the patients were followed up via a telephone call introducing risk of recall bias.
			Infection	Comparison between closure types - Incidence of post-operative infection in SL group = 2/59 (3.4%) - Incidence of post-operative infection in DL group = 4/60 (6.7%) Difference not statistically significant. Comparison between incidence of infection according to operating surgeon - Patient group done by one physical assistant = 6/119 (5%) - Patient group done by surgical residents = 15/120 (13%) *p* < 0.05	
	Scandinavian Cardiovascular Journal, Norway, Prospective randomised trial (Level 1b)		Oedema/erythema/haematoma/seroma	Not assessed.	
			Length of admission	Not assessed.	
			Other	No further assessment.	
Single-layer versus multiple-layer closure of leg wounds after long saphenous vein harvest: a prospective randomized trial [13]	Zafar et al. (2005)	n = 78 - Single layer over a suction drain (SL) = 44 - Double layer (DL) = 34 NB. From the 78 patients recruited, 8 patients had the long saphenous harvested from both legs and therefore 86 legs were studied.	Length of incision	- SL = 51.2 cm (Mean) - DL = 48.7 cm (Mean) Difference not statistically significant (*p* = 0.49).	Minimisation was used to allow for a method of randomisation which accounts for the imbalance between patients. The statistical analysis method is clearly delineated. No declaration of conflict of interest. Discrepancy in the gender of patients was present (67 male and 11 female patients), but diabetics were equally distributed amongst the two groups. The above discussed discrepancies may be a source of selection bias in this study. NB. a lower ASEPSIS score indicates better healing.
			Infection	ASEPSIS Wound score (Mean) - SL = 4.38 - DL = 8.24 *p* = 0.001	
	The Annals of Thoracic surgery, United Kingdom, Prospective randomised trial (Level 1b)		Oedema/erythema/haematoma/seroma	Oedema - SL = 9.1%. - DL = 82.4% *p* < 0.001	
			Length of admission	Not assessed.	
			Other	Antibiotic requirement - SL = 18.2% - DL = 25.7% Level of statistical significance not mentioned.	
Should we stitch the subcutaneous fat layer following saphenous vein excision for coronary revascularization? [16]	El Gamel et al. (1994)	Group 1 - n = 100 [Below knee saphenectomy of both legs of the same patient with one leg randomly assigned to no fat suture (1A–100 wounds) and the contralateral leg of the same patient to fat suturing (1B–100 wounds). As a result of both legs of every patient being used, group 1 had a total of 200 leg wounds]. Group 2 - n = 200 [Above knee/thigh saphenectomy with random allocation to either no fat suture (2A–100 wounds) or a fat suture (2B–100 wounds)].	Length of incision	Not assessed.	Randomisation method not defined. However, the statistical analysis method is clearly delineated. No declaration of conflict of interest. Group 2 had 200 patients compared with group 1 which had 100 patients in total. Group 1 displayed gender disparity with 91 male and 9 female patients. There was also a gender disparity in group 2 where 30% of the participants were female. Further, discrepancy was also displayed in other demographic domains (though no statistical analysis carried out): - Peripheral vascular disease - Group 1 (15%) compared with group 2 (29%). - Diabetes - Group 1(16.5%) compared with group 2 (39%). The above discussed discrepancies may be a source of selection bias in this study. Group 2 participant number is mismatched with the breakdown; the total is indicated as 100, yet the breakdown shows that there are 69 male and 30 female patients adding up to a total of 99 with no account of/explanation for the inconsistency. Further, the recruitment of one group of patients from the UK and the other group from the USA introduces risk of sampling bias.
			Infection	- Group 1A (No fat suture) - 1/200 wounds were complicated with infection. - Group 1B (Fat sutured) - 1/200 wounds were complicated with infection. - Group 2A (No fat suture) - 2/200 wounds were complicated with infection. - Group 2B (Fat sutured) - 2/200 wounds were complicated with infection. Statistical significance not assessed, though note that the incidence is the same in both subgroups.	
	European Journal of Cardio-Thoracic Surgery, United Kingdom and United States of America, Prospective randomised trial (Level 1b)		Oedema/erythema/haematoma/seroma	- Group 1A (No fat suture) - 2/200 complicated by a haematoma. - Group 1B (Fat sutured) - 1/200 complicated by a haematoma. - Group 2A (No fat suture) - 3/200 complicated by a haematoma. - Group 2B (Fat sutured) - 5/200 complicated by a haematoma. All haematomas in the fat suture subset of group 2 required surgical evacuation and plastic surgical intervention for skin edge necrosis, compared to 1 case in the no fat suture group (*p* > 0.025).	
			Length of admission	Not assessed.	
			Other	Wound complication rate - 3% in Group 1B compared to 2% in Group 1A (Not statistically significant). - The overall wound complication rate of group 2 was the same for both subsets. Closure time - Group 1A (No fat suture) was 20 minutes compared with 1B which was 40 minutes. - Group 2A (No fat suture) was 21 minutes compared with 2B which was 38 minutes.	
A comparison of single-layer versus multi-layer closure of the leg wound following long saphenous vein harvest for coronary artery bypass graft surgery: a prospective randomised controlled trial [14] (Article in Persian)	Nouraei et al. (2010)	n = 80 - Single layer and Haemovac (SL) = 40 - Multiple layer (ML) = 40	Findings at day 2 post-operatively	Higher incidence of haematoma in the ML group: SL (2.5%) vs ML (45%) - *p* < 0.001	It is mentioned that simple randomisation was utilised, though there is no further elaboration on this; the method of double blinding is explained. Further, the statistical analysis method is clearly delineated. No declaration of conflict of interest. Over 70-year olds, diabetics, BMI >30 and urgent cases excluded. No mention of how surgical site infection was managed. Apart from the abstract, the article is in Persian which was translated by Pedram Panahi (First author) for inclusion in the table.
			Findings at day 14 post-operatively	In the multiple-layer closure group, there was a higher incidence of: - Ecchymosis (37.5% of SL vs 67.5% of ML - *p* = 0.007) - Haematoma (2.5% of SL vs 47.5% of ML - *p* = 0.000) - Seroma discharge (22.5% of SL vs 47.5% of ML - *p* = 0.018) - Infection (12.5% of SL vs 37.5% of ML - *p* = 0.009) - Skin necrosis (2.5% of SL vs 17.5% of ML - *p* = 0.018) - Pain (35% of SL vs 77.5% of ML - *p* = 0.000). - Saphenous nerve paraesthesia (17.5% of SL vs 32.5% of ML - *p* = 0.119/NS)	
	Journal of Mazandaran University of Medical Sciences, Iran, Prospective randomised trial (Level 1b)		Findings at day 21 post-operatively	In the multiple-layer closure group, there was a higher incidence of: - Ecchymosis (27.5% of SL vs 55% of ML - *p* = 0.012) - Haematoma (5% of SL vs 40% of ML - *p* = 0.000) - Seroma discharge (17.5% of SL vs 40% of ML - *p* = 0.025) - Infection (17.5% of SL vs 40% of ML - *p* = 0.025) - Skin necrosis (7.5% of SL vs 17.5% of ML - *p* = 0.171/NS) - Pain (20% of SL vs 77.5% of ML - *p* = 0.000) - Saphenous nerve paraesthesia (20% of SL vs 35% of ML - *p* = 0.131/NS)	
			Length of admission	Not assessed.	
			Other	No further assessment.	

Appendix 1. Best evidence articles (*p* = level of significance; NS = Not statistically significant).
